# Impact of chemotherapy on lymphocytes and serological memory in recovered COVID-19 patients with acute leukemia

**DOI:** 10.7150/jca.53863

**Published:** 2021-03-01

**Authors:** Wei Liu, Ziping Li, Wenjuan He, Dan Yu, Ping Wang, Li Cai, Peng Yang, Xuexing Chen, Xiaoping Zhang, Hao Zhou

**Affiliations:** 1 Center for Stem Cell Research and Application, Union Hospital, Tongji Medical College, Huazhong University of Science and Technology, Wuhan 430022, China.; 2 Institute of Hematology, Union Hospital, Tongji Medical College, Huazhong University of Science and Technology, Wuhan 430022, China.; 3 Department of Hematology, Wuhan No.1 Hospital, Tongji Medical College, Huazhong University of Science and Technology, Wuhan 430030, China.; 4 Department of Clinical Laboratory, Union Hospital, Tongji Medical College, Huazhong University of Science and Technology, Wuhan 430022, China.; 5 Department of Breast and Thyroid Surgery, Union Hospital, Tongji Medical College, Huazhong University of Science and Technology, Wuhan 430022, China.

**Keywords:** COVID-19, leukemia, chemotherapy, lymphocytes, antibody, immune memory

## Abstract

Chemotherapy is the major method of treatment for acute leukemia to date, while intensive chemotherapy may impair immunity. We previously reported that leukemia patients were more susceptible to COVID-19 than the overall population. However, for COVID-19 recovered patients with leukemia, the impacts of intensive chemotherapy on the immune memory of COVID-19 are unknown. This study characterized the changes in immune cells and SARS-CoV-2 antibodies in acute leukemia patients, who underwent chemotherapy after recovering from COVID-19. The study enrolled three groups of individuals. One group was a total of three acute leukemia patients, who recovered well from COVID-19 before the last cycle of chemotherapy. The other two groups were six COVID-19 recovered healthy people, and six normal uninfected healthy people, respectively. Levels of B cells, T cells, and NK cells in peripheral blood were analyzed by multiparameter flow cytometry. Besides, the SARS-CoV-2 antibodies were monitored. The results showed that B cells were severely decreased after chemotherapy, especially memory B cells. Most of the T cells and NK cells showed only minor changes after chemotherapy, except for γδ T cells. The serum levels of SARS-CoV-2 antibodies were not significantly affected after chemotherapy in two leukemia patients. However, interestingly, one leukemia patient's SARS-CoV-2 IgM showed dramatically increase, suggesting possible loss of serological memory after chemotherapy. These findings raised the concern for the stability of immune memory against SARS-CoV-2 during chemotherapy and the choice of anti-leukemia treatment in the COVID-19 pandemic.

## Introduction

Acute leukemia (AL) is a collection of heterogeneous malignant neoplastic disease, which is distinct from solid tumors largely by the presence of symptoms related to bone marrow failure or suppression 1. The anti-leukemia treatment severely affects the immune system, causing a massive depletion of cells in the immune system. Besides, previously attained immunity against various infectious antigens can be attenuated during chemotherapy, and some leukemia patients show an impaired response to immunizations 2.

Infections are the major complication in the management of hematologic malignancies. We previously reported that leukemia patients were more susceptible to COVID-19 than the general population and tend to have a worse clinical outcome, owing to their immunosuppressed status caused by both leukemia and chemotherapy 3. Given the worldwide prevalence of leukemia and the high transmissibility of SARS-CoV-2, an intentional postponing of chemotherapy for stable cancer has been considered in pandemic areas 4.

The COVID-19 pandemic in China has been well controlled presently. As a beneficial consequence, some leukemia patients who have recovered from COVID-19 and got protective SARS-CoV-2 antibodies begin to consider undergoing chemotherapy, given the rapid progression feature of acute leukemia. However, it is unclear about the changes of lymphocytes and whether the protective SARS-CoV-2 antibodies are stable in these COVID-19 recovered leukemia patients after chemotherapy. Also, it is obscure for the subsequent use of the COVID-19 vaccine soon in uninfected cancer patients. Additionally, changes in adaptive immune cells after chemotherapy in leukemia may have implications for the success of immunotherapy.

In the present study, we evaluated the levels of B, T, and natural killer (NK) cells in COVID-19 recovered leukemia patients who received chemotherapy, as well as COVID-19 recovered healthy people and normal uninfected healthy people. For this purpose, we measured the numbers of various lymphocyte subsets in peripheral blood using multiparameter flow cytometry. Moreover, the SARS-CoV-2 antibodies were monitored between the COVID-19 recovered leukemia group and the recovered healthy group. We also traced antibodies against the hepatitis B virus (HBV) as an indicator in the leukemia group since the national HBV vaccination policy of China 5. The aim is to explore the status of immune cells and whether the serological memory stable after chemotherapy in COVID-19 recovered leukemia patients. These findings raised the concern for the stability of immune memory against SARS-CoV-2 during chemotherapy and the choice of anti-leukemia treatment in the COVID-19 pandemic.

## Materials and Methods

### Study design and participants

The study included three groups of people: three COVID-19 recovered leukemia patients; six COVID-19 recovered healthy people; six normal uninfected healthy people. Peripheral blood samples were collected. The criteria for infection and recovery of COVID-19 were defined according to the diagnostic criteria of “COVID-19 diagnosis and treatment Plan (trial seventh edition)” 6. In addition, the SARS-CoV-2 nucleic acid tests of the COVID-19 recovered patients remained negative throughout the study. Ethics approval was obtained from the Ethics Committee of Wuhan Union Hospital, Tongji Medical College, Huazhong University of Science and Technology, Wuhan, China. Written informed consents were obtained from each study participant.

### Reagents and instruments

Detection reagents are as follows: CD3, CD4, CD5, CD8, CD10, CD11b, CD11c, CD14, CD16, CD19, CD20, CD21, CD23, CD24, CD25, CD27, CD33, CD38, CD45, CD45RA, CD56, CD80, CD123, Foxp3, IL-17, HLA-DR, TCRαβ, TCRγδ, The above reagents were purchased from BD Corporation (San Jose, CA, USA), Beckman Coulter (Indianapolis, Indiana, USA) and eBioscience (San Diego, CA, USA). Testing instrument is BD FACSCanto II. Analysis software is CellQuest. The COVID-19 was confirmed with nucleic acid detection kit (Shanghai bio-germ Medical Technology, Shanghai, China). The SARS-CoV-2 antibodies (IgM and IgG) were detected using a sandwich enzyme linked immunosorbent assay (ELISA kits, Livzon Inc, Zhuhai, China, lot number of IgM: 20200308, IgG: 20200308).

### Flow cytometry

The percentages of lymphocyte subsets were determined by flow cytometry. Briefly, cells were acquired on a FACSCanto II flow cytometer before analysis in CellQuest (Becton Dickinson). For all lymphocyte subsets, percentages were determined and absolute numbers were calculated. Lymphocytes were identified using forward scatter and side scatter. To divide lymphocytes into different subsets, established standard and well-described gating strategies were used. B lymphocytes were identified by the presence of CD19 and were further divided into naïve B cells, memory B cells, transitional B cells, and plasma cells. T lymphocytes were defined by positivity for CD8 or CD4 were subdivided into helper/induced T cells, inhibited/cytotoxic T cells, Tregs, Th17 cells, αβ T cells, and γδ T cells. NK-like T cells were defined as CD56+ CD3+. NK lymphocytes were detected as (CD56+/CD16+) CD3-. Detailed information on fluorochrome-antibody conjugates as well as the specific gating strategies for these lymphocytes and their subsets can be found in Supplementary (Table S1, S2, and Figure S1-6).

### Statistical analysis

Results are given as median values with interquartile ranges for descriptive analysis if not otherwise indicated. Mann-Whitney U test for associated samples was used for all statistical tests (two-tailed) to detect different numbers of lymphocytes and their subsets. Data were analyzed with SPSS for Windows (IBM SPSS Statistics 25, Armonk, New York, USA). A two-tailed P value <0.05 was regarded as statistically significant.

## Results

### Patient demographics

Demographics and clinical information of the three COVID-19 recovered leukemia patients are summarized in Table 1. The average age of leukemia patients was 45 years (range 36-57) with a median age of 43 years. The common symptom of COVID-19 onset was fever. Two patients were in complete remission (CR) and one was in partial remission (PR) following chemotherapy, and the average time elapsed from nucleic acid turns negative to chemotherapy treatment was 8 weeks (range 4-15 weeks). Moreover, the demographics of six recovered healthy people and normal people were matched with acute leukemia patients.

### Lymphocyte subsets show differential depletion and recovery after chemotherapy

To better understand the effects on the lymphocyte subsets of COVID-19 recovered leukemia patients. After 2 weeks of chemotherapy, flow cytometry was performed on COVID-19 recovered leukemia patients. To understand the changes of lymphocyte subsets, we analyzed peripheral blood of COVID-19 recovered leukemia group, and found that the proportion of T cells and NK cells was in the normal range. And the proportion of B cells is significantly reduced. The absolute number of B cells was also significantly reduced. Meanwhile, we also collected samples from the recovered healthy group and normal healthy group. We also performed flow cytometry on recovered healthy people and normal healthy people, and found that the proportion of T cells, NK cells, and B cells of COVID-19 recovered healthy people and, normal uninfected healthy people were basically within the normal range (the detailed data was shown in Table 2).

### Changes of B cell subsets

We found that the proportion of B lymphocytes varied greatly by flow detection, so we performed further flow analysis on the B cell subsets. CD19+ B cells were analyzed as: naïve B lymphocytes (CD10- CD19+ CD20+ CD27- CD38-/+ CD45+), memory B lymphocytes (CD10- CD19+ CD20+ CD27+ CD38-/+ CD45+), transitional B lymphocytes (CD10dim CD19+ CD20+ CD27- CD38high CD45+), and plasma cell (CD10- CD19+ CD20+ CD27+ CD38high CD45dim). We detected three COVID-19 recovered leukemia patients, as well as COVID-19 recovered healthy people and normal uninfected healthy people. We analyzed the general proportion of each subset of B cells and found that the B cell subsets in COVID-19 recovered healthy people and normal healthy people were the same, while the B cells in COVID-19 recovered leukemia patients were different from the first two groups. The amount of memory B cells in COVID-19 recovered leukemia patients decreased dramatically. The plasma cells of the three groups were also analyzed, nevertheless the results showed no significant difference.

### Changes of T cell subsets

We also examined related T cell subsets as follows: CD3+CD4+ helper/induced T cells, CD3+CD8+ inhibited/cytotoxic T cells, Treg cells, Th17 cells, αβ T cells, γδ T cells. We examined COVID-19 recovered leukemia patients, COVID-19 recovered healthy people and normal uninfected healthy people. The results showed that the T cell subsets of the recovered healthy people were the same as those of the normal healthy people, with no significant difference and all within the normal range. Compared with the first two groups, the proportion of γδ T cells in COVID-19 recovered leukemia patients was extremely low and almost invisible, which was much lower than that of the recovered healthy group and normal healthy group.

### Changes of NK cell subsets

NK cells in COVID-19 recovered leukemia patients, COVID-19 recovered healthy people and, normal uninfected healthy people were in the normal range, and showed no significant changes.

### Changes of serum antibody level before and after chemotherapy

The level of SARS-CoV-2 antibodies in one leukemia patient was seemingly reversed class-switch (from anti-SARS-CoV-2 IgG to IgM) after chemotherapy, while the changes of serum antibodies of the other two leukemia patients were consistent with those of COVID-19 recovered healthy people. We found that the HBV antibodies remained stable before and after chemotherapy. The detailed data was shown in the Supplementary part (Table S3). Additionally, the level of SARS-CoV-2 antibodies in COVID-19 recovered healthy people was shown in the Supplementary part (Table S4).

## Discussion

In the present study, we focused on the impact of chemotherapy on the lymphocyte subsets and SARS-CoV-2 antibodies levels in COVID-19 recovered leukemia patients. We found that B cells were severely affected in COVID-19 recovered leukemia group, especially memory B cells. While most of the T cells and NK cells were showed only minor changes in the COVID-19 recovered leukemia group, except for γδ T cells. Also, the number of various lymphocyte subsets showed no significant difference and within the normal range in the two control groups. Interestingly, one leukemia patient whose SARS-CoV-2 antibodies showed seemingly reversed class-switch after chemotherapy, while serum levels of SARS-CoV-2 antibodies were not significantly affected after the intensive chemotherapy in the other two leukemia patients.

In the context of the COVID-19 pandemic, it is important to possess an immune system that can defend against SARS-CoV-2, especially B cells that could produce neutralizing antibodies and differentiate into memory B cells to prevent re-infection. The total B lymphocytes in COVID-19 recovered leukemia patients were significantly decreased, while the number of B lymphocytes in COVID-19 recovered healthy people is no different from normal healthy people. Previous studies reported that lymphocytes in post-chemotherapy are significantly lower than in pre-chemotherapy in acute leukemia patients 7,8. Specifically, in our cases, the memory B cells in the COVID-19 recovered leukemia patients reduced significantly. We speculate that the dramatic decline of B lymphocytes might be caused by chemotherapy, and B cells may be more susceptible to chemotherapy or slower in recovery, comparing with T cells and NK cells.

Interestingly, in one of the leukemia patients, the dynamic changes of SARS-CoV-2 antibodies showed that the immune memory to COVID-19 was seemingly lost. With the national hepatitis B virus (HBV) vaccination policy of China, this patient has been previously vaccinated with the HBV vaccine, and the HBs antibodies of this patient were kept stable over time. The re-appearance of high titers of SARS-CoV-2 IgM antibody, in this case, illustrates the possibility that the loss of serological memory to SARS-CoV-2 after chemotherapy. Since depletion of lymphocytes and protective antibodies have been identified in post-chemotherapy patients 9, the chemotherapy may be a causal factor for the defect of memory B cells and serological memory to SARS-CoV-2 in our case. Nevertheless, the immune memory against SARS-CoV-2 seemed to be more fragile than that against HBV. During the COVID-19 pandemic, given these, intensive chemotherapy should be administered with caution and the antibodies should be monitored in cancer patients with past SARS-CoV-2 infection.

Researchers worldwide are working around the clock to find a vaccine against SARS-CoV-2. Previous studies have found that patients exhibit reduced serum titers of vaccine-specific antibodies against tetanus, pneumococcal, and other antigens for more than half a year after chemotherapy 10,11. Besides, several studies presented that the recovery of B cells after chemotherapy lasts for a long time 12,13. Considering the serological memory loss of the COVID-19 in our study, for cancer patients who have undergone chemotherapy, evaluations of the patients' immune response are required before and after the administration of the vaccine against SARS-CoV-2. Moreover, it also should be clarified whether against SARS-CoV-2 revaccination is needed in cancer patients who received chemotherapy.

Comparing with the two non-leukemia groups, the γδ T cells in leukemia patients were extremely low and almost invisible, while other T cell subsets were grossly normal. In general, γδ T cells may be considered a component of adaptive immunity in that they could participate in developing a memory phenotype, notably in building memory T cells 14. The number of γδ T cells was almost invisible in the leukemia patient who seems to lose serological memory, while the number of γδ T cells in the other two patients is relatively countable. Consequently, we speculate the deficiency of γδ T cells in COVID-19 recovered leukemia patients may partially account for the loss of immune memory. Previous research demonstrated that γδ T cells subset-targeting could be beneficial to enhance or inhibit the outcome of adaptive immune responses against cancer 15, suggesting that these cells would be effective in cancer therapy.

Our study still had some limitations. Firstly, the sample size was too small to derive any firm conclusions. Secondly, we were not able to analyze the effect of different chemotherapy regimens on lymphocytes because the regimens are highly heterogeneous and the sample size was so small for scientific statistical analysis. Thirdly, for making a description of regeneration of B cells, T cells, and NK cells after cessation of chemotherapy, our data do not reflect a sufficient time period.

## Supplementary Material

Supplementary figures and tables.

## Figures and Tables

**Table 1:** Baseline characteristics of recovered COVID-19 patients with acute leukemia

Characteristics	Patient 1	Patient 2	Patient 3
Age (y)	43	36	57
Gender (M/F)	F	F	M
Smoking (Y/N)	N	N	Y
Comorbidities	none	none	chronic hepatitis
**COVID-19**			
Symptoms of onset	fever	fever, cough, vomit	fever, cough, fatigue
Severity	severe	severe	moderate
Weeks from nucleic acid turns negative to chemotherapy	4	15	4
**Hematological malignancies**			
Diagnosis	AML	ALL	AML
Weeks since initial diagnosis	5	32	23
Chemotherapy regimens	IA	hyperCVAD Course A	IA
Remission status	CR	CR	PR

Abbreviations. AML: acute myeloid leukemia, ALL: acute lymphoblastic leukemia, IA: idarubicin cytarabine, hyper CVAD Course A: hyper fractionated cyclophosphamide, vincristine, doxorubicin, and dexamethasone, CR: complete remission, PR: partial remission.

**Table 2:** Changes of lymphocyte subsets after chemotherapy

Lymphocyte subsets	Patient 1	Patient 2	Patient 3	*P value*	Recovered healthy	Normal healthy	*P value*
				(patients vs. recovered healthy)			(recovered healthy vs normal healthy)
**B lymphocytes (/ul)**							
B lymphocytes	9.95	64.67	85.58	<0.05	160.05 [147.08; 224.46]	237.82 [179.46; 286.22]	n.s.
Naïve B cells	6.44	44.35	53.53	<0.05	115.55 [98.66; 169.82]	180.99 [116.37; 204.55]	n.s.
Memory B cells	3.01	18.70	27.96	<0.05	42.15 [36.30; 60.04]	52.84 [37.9; 68.5]	n.s.
Transitory B cells	0.48	1.02	0.75	n.s.	2.35 [2.04; 4.52]	3.99 [3.01; 5.47]	n.s.
Plasma cells	2.48	2.27	3.10	n.s.	2.88 [2.30; 4.66]	4.23 [2.85; 6.16]	n.s.
**T lymphocytes (/ul)**							
T lymphocytes	637.59	626.93	694.07	n.s.	842.35 [684.71; 909.05]	830.02 [677.56; 930.11]	n.s.
Helper/induced T cells	334.15	249.88	407.45	n.s.	585.40 [411.05; 622.94]	573.47 [436.77; 648.42]	n.s.
Inhibit/cytotoxic T cells	252.55	309.17	221.97	n.s.	246.75 [227.44; 301.32]	230.77 [201.52; 284.60]	n.s.
CD4+CD8+T cels	4.19	4.36	3.45	n.s.	4.50 [2.75; 6.28]	2.35 [1.79; 5.41]	n.s.
CD4:CD8 ratio	1.32	0.80	1.84	n.s.	2.37 [0.91; 3.22]	2.49 [1.08; 3.50]	n.s.
Tregs	15.67	23.51	19.28	n.s.	28.91 [17.91; 54.85]	42.77 [20.44; 61.31]	n.s.
Th17	18.93	27.70	24.19	n.s.	40.47 [26.63; 48.30]	36.66 [21.02; 43.64]	n.s.
αβ T cells	636.23	617.39	683.50	n.s.	809.63 [671.80; 905.37]	800.41 [663.28; 925.60]	n.s.
γδ T cells	0.05	7.31	9.42	<0.01	31.95 [19.33; 40.51]	30.55 [19.72; 38.66]	n.s.
**NK cells (/ul)**							
NK cells	117.81	131.35	126.38	n.s.	224.50 [166.58; 322.84]	238.5 [161.30; 312.08]	n.s.
NK/T cells	14.25	19.38	15.77	n.s.	20.25 [15.50; 24.49]	19.27 [14.46; 25.62]	n.s.

Continuous variables are reported as median [interquartile range]. n.s. means not significant.

## Abbreviations

AL: acute leukemia
NK: natural killer
HBV: hepatitis B virus
CR: complete remission
PR: partial remission
AML: acute myeloid leukemia
ALL: acute lymphoblastic leukemia
IA: idarubicin cytarabine, hyper CVAD Course A, hyper fractionated cyclophosphamide, vincristine, doxorubicin, and dexamethasone

## References

[1] Arber DA, Orazi A, Hasserjian R (2016). The 2016 revision to the World Health Organization classification of myeloid neoplasms and acute leukemia. Blood.

[2] Goswami M, Prince G, Biancotto A (2017). Impaired B cell immunity in acute myeloid leukemia patients after chemotherapy. J Transl Med.

[3] He W, Chen L, Chen L (2020). COVID-19 in persons with haematological cancers. Leukemia.

[4] Tang LV, Hu Y (2020). Poor clinical outcomes for patients with cancer during the COVID-19 pandemic. The Lancet Oncology.

[5] Renelus BD, Jamorabo DS, Mohanty SR (2019). Global Elimination of Chronic Hepatitis. The New England journal of medicine.

[6] National Health Commission of People's Republic of China. Diagnosis and treatment of pneumonia caused by novel coronavirus (trial seventh edition). (.

[7] Saghafian-Hedengren S, Söderström I, Sverremark-Ekström E (2018). Insights into defective serological memory after acute lymphoblastic leukaemia treatment: The role of the plasma cell survival niche, memory B-cells and gut microbiota in vaccine responses. Blood Rev.

[8] Waidhauser J, Schuh A, Trepel M (2020). Chemotherapy markedly reduces B cells but not T cells and NK cells in patients with cancer. Cancer immunology, immunotherapy: CII.

[9] Litterman AJ, Zellmer DM, Grinnen KL (2013). Profound impairment of adaptive immune responses by alkylating chemotherapy. Journal of immunology (Baltimore, Md: 1950).

[10] Fioredda F, Giacchino M, Castagnola E (2005). Assessment of humoral immunity to poliomyelitis, tetanus, hepatitis B, measles, rubella, and mumps in children after chemotherapy. Cancer.

[11] Nazi I, Kelton JG, Larché M (2013). The effect of rituximab on vaccine responses in patients with immune thrombocytopenia. Blood.

[12] Verma R, Foster RE, Horgan K (2016). Lymphocyte depletion and repopulation after chemotherapy for primary breast cancer. Breast Cancer Res.

[13] van Tilburg CM, van Gent R, Bierings MB (2011). Immune reconstitution in children following chemotherapy for haematological malignancies: a long-term follow-up. British Journal of Haematology.

[14] Sheridan BS, Romagnoli PA, Pham QM (2013). γδ T cells exhibit multifunctional and protective memory in intestinal tissues. Immunity.

[15] Vitiello GA, Miller G (2020). Targeting the interleukin-17 immune axis for cancer immunotherapy. The Journal of experimental medicine.

