# Supplementary material for: Impact of chemotherapy on lymphocytes and serological memory in recovered COVID-19 patients with acute leukemia
Source: J Cancer. 2021 Mar 1;12(8):2450–5. doi: 10.7150/jca.53863 (PMC7974873; doi:10.7150/jca.53863)
Supplement: Supplementary file 1 — Supplementary figures and tables. [file jcav12p2450s1.pdf]

Table S1: Fluorochrome-antibody conjugates.

Antibodies	Clone	Corporation
CD3	SK7	BD
CD4	SK3	BD
CD5	BL1a	Beckman Coulter
CD8	SK1	BD
CD10	HI10a	BD
CD11b	ICRF44	BD
CD11c	S-HCL-3	BD
CD14	MφP9	BD
CD16	3G8	BD
CD19	SJ25C1	BD
CD20	L27	BD
CD21	B-ly4	BD
CD23	9P25	Beckman Coulter
CD24	SN3	eBioscience
CD25	2A3	BD
CD27	L128	BD
CD33	p67-6	BD
CD38	HIT2	BD
CD45	HI30	BD
CD45RA	L48	BD
CD56	NCAM16.2	BD
CD80	L307.4	BD
CD123	9F5	BD
Foxp3	259D/C7	BD
IL-17	N49-653	BD
HLA-DR	L243	BD
TCRαβ	WT31	BD
TCRγδ	11F2	BD

Table S2. Lymphocytes Immunophenotype.

Lymphocyte subsets	Immunophenotype
**B lymphocytes**	
B lymphocytes	$CD19^{+}$
Naïve B cells	$CD10^{-}CD19^{+}CD20^{+}CD27^{-}CD38^{-/+}CD45^{+}$
Memory B cells	$CD10^{-}CD19^{+}CD20^{+}CD27^{+}CD38^{-/+}CD45^{+}$
Transitory B cells	$CD10^{dim}CD19^{+}CD20^{+}CD27^{-}CD38^{hi}CD45^{+}$
Plasma cells	$CD10^{-}CD19^{+}CD20^{+}CD27^{++}CD38^{hi}CD45^{dim}$
**T lymphocytes**	
T lymphocytes	CD3+
Helper/induced T cells	CD3+CD4+
Inhibit/cytotoxic T cells	CD3+CD8+
CD4+CD8+T lymphocytes	CD4+CD8+
CD4:CD8 ratio	CD4/CD8
Tregs	CD4+CD25+Foxp3+
Th17	CD4+IL-17+
γδ T cells	CD3+TCRγδ+
αβ T cells	CD3+TCRαβ+
**NK cells**	
NK cells	CD3-(CD56+/CD16+)
NK/T cells	CD56+CD3+

Table S3. Changes of serum antibody level before and after chemotherapy.

Antibodies	Patient 1		Patient 2		Patient 3	
	**before chemotherapy**	**after chemotherapy**	**before chemotherapy**	**after chemotherapy**	**before chemotherapy**	**after chemotherapy**
**anti-SARS-CoV-2**						
**IgM (AU/ml)**	< 10	268.06	< 10	< 10	< 10	< 10
**IgG (AU/ml)**	12.37	11.62	35.61	24.86	52.90	37.27
**anti-HBs IgG (mIU/ml)**	30.14	48.69	102.59	96.02	5.68	7.57

"< 10" means a negative result.

Antibodies	Patient 1		Patient 2		Patient 3		Patient 4		Patient 5		Patient 6	
	on admission	on discharge	on admission	on discharge	on admission	on discharge	on admission	on discharge	on admission	on discharge	on admission	on discharge
**anti-SARS-CoV-2**												
**IgM (AU/ml)**	123.41	12.76	37.8	<10	25.63	<10	127.37	50.62	98.33	32.71	207.64	98.32
**IgG (AU/ml)**	70.36	150.32	102.73	80.53	573.24	106.58	113.29	92.27	200.75	102.36	50.71	48.67
**anti-HBs IgG (mIU/ml)**	92.71	NA	79.33	NA	42.12	NA	129.84	NA	23.78	NA	140.31	NA

Table S4. Changes of serum antibody level in COVID-19 recovered healthy people.

“<10” means a negative result.

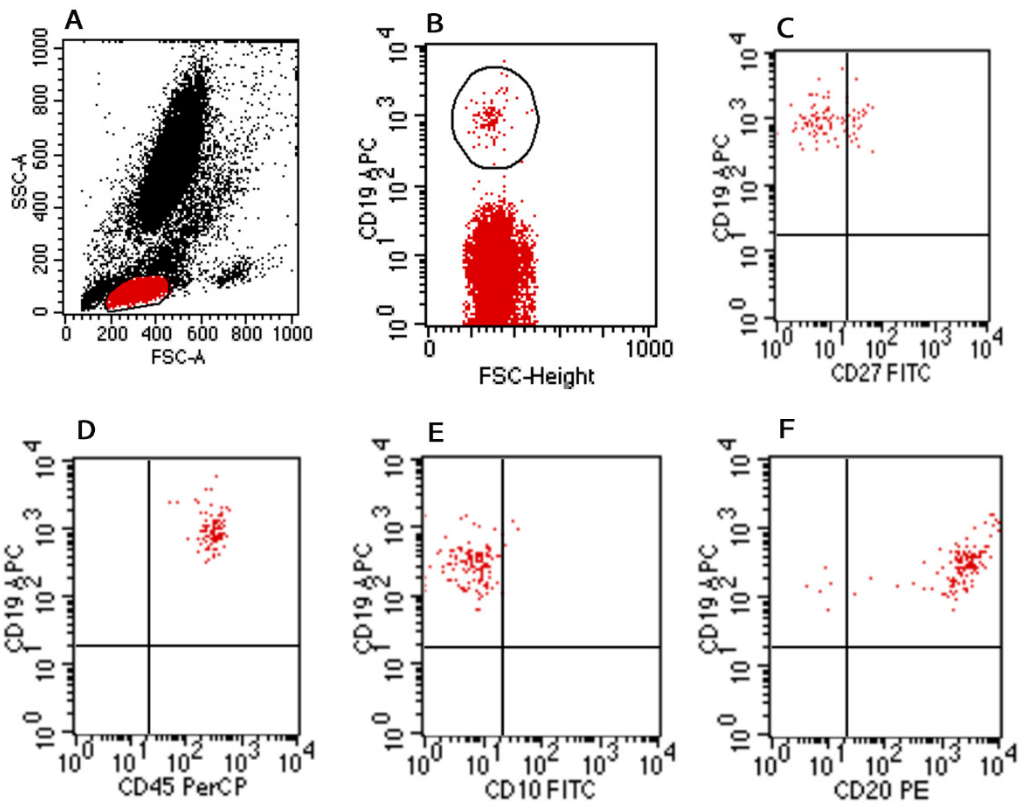

Figure S1. The data of the above pictures are from the COVID-19 recovered leukemia patients. (**A**) Lymphocytes were identified using forward and side scatter. (**B**) B lymphocytes were defined by the presence of CD19. (**C-F**) Further divided into naïve B lymphocytes (CD10-CD19+ CD20+ CD27-CD38-/+ CD45+), memory B lymphocytes (CD10- CD19+ CD20+ CD27+ CD38-/+CD45+), transitional B lymphocytes (CD10dimCD19+CD20+CD27-CD38hiCD45+) subsets. Since B lymphocytes are significantly reduced, not enough cell signals could be obtained.

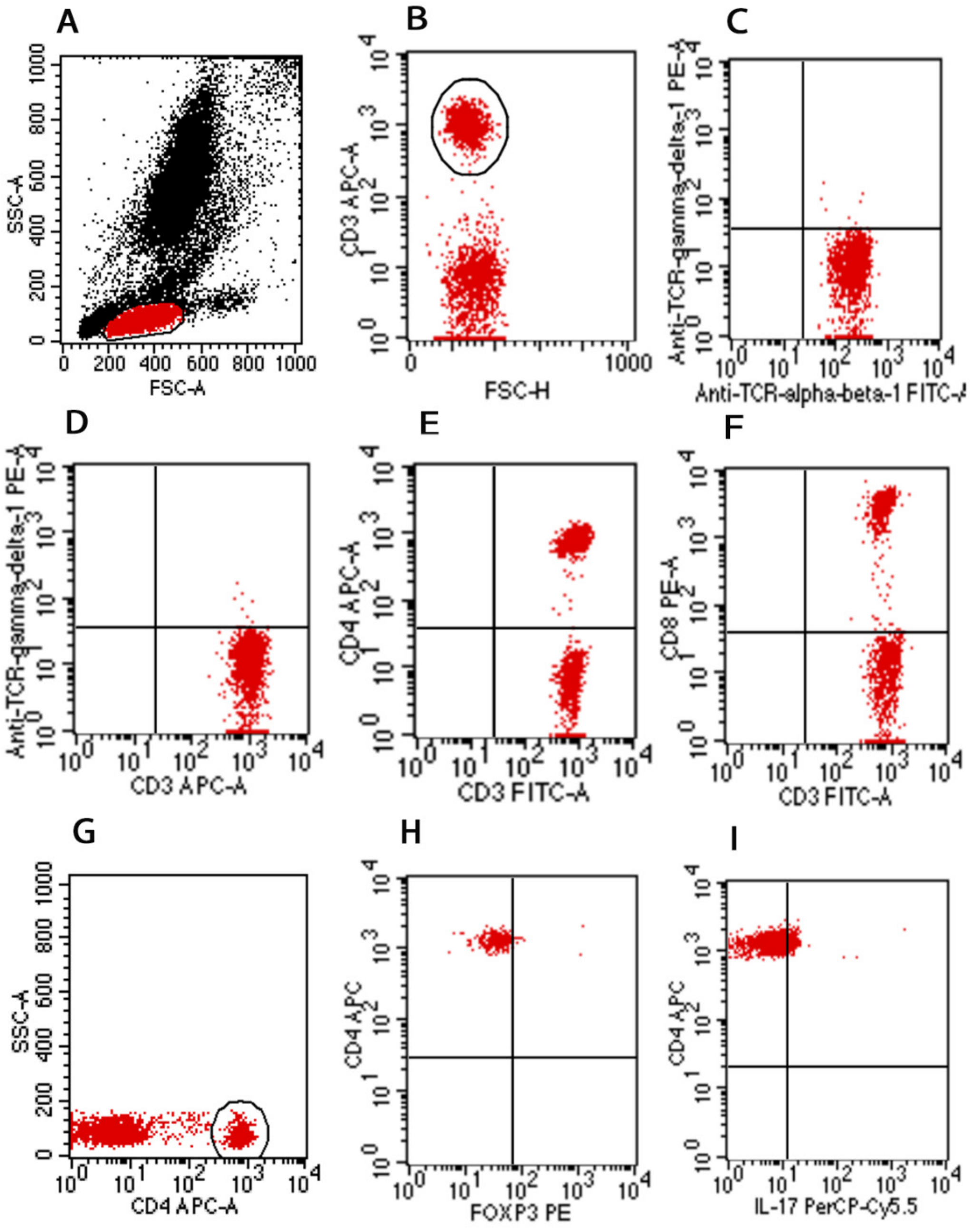

Figure S2. The data of the above pictures are from the COVID-19 recovered leukemia patients. **(A)** Lymphocytes were identified using forward and side scatter. **(B)** T lymphocytes were defined by the presence of CD3. **(C-D)** Further divided into αβ T cells, γδ T cells, **(E)** CD3+CD4+ helper/induced T cells. **(F)** CD3+CD8+ inhibited/cytotoxic T cells. **(G)** CD4+T lymphocytes were defined and further divided into **(H)** Tregs and **(I)** TH17 cells.

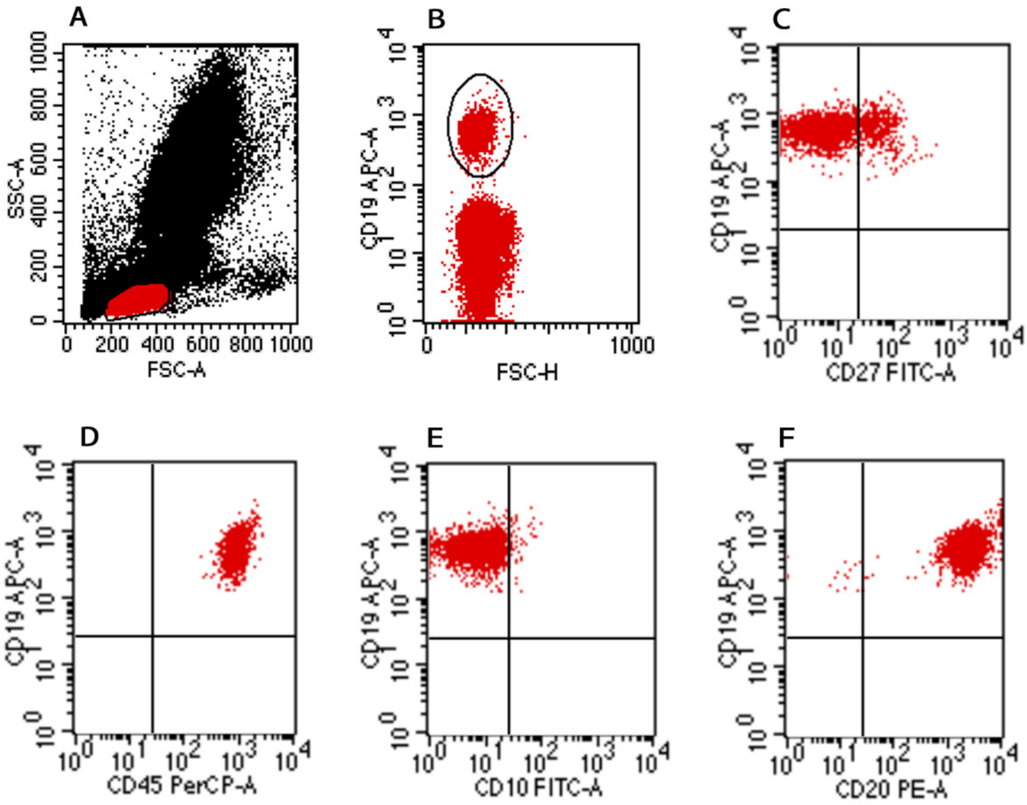

Figure S3. The data of the above pictures are from the COVID-19 recovered healthy people. **(A)** Lymphocytes were identified using forward and side scatter.**(B)** B lymphocytes were defined by the presence of CD19, **(C-F)** and further divided into naïve B lymphocytes (CD10-CD19+ CD20+ CD27-CD38-/+ CD45+), memory B lymphocytes (CD10- CD19+ CD20+ CD27+ CD38-/+CD45+), transitional B lymphocytes (CD10dimCD19+CD20+CD27-CD38hiCD45+) subsets.

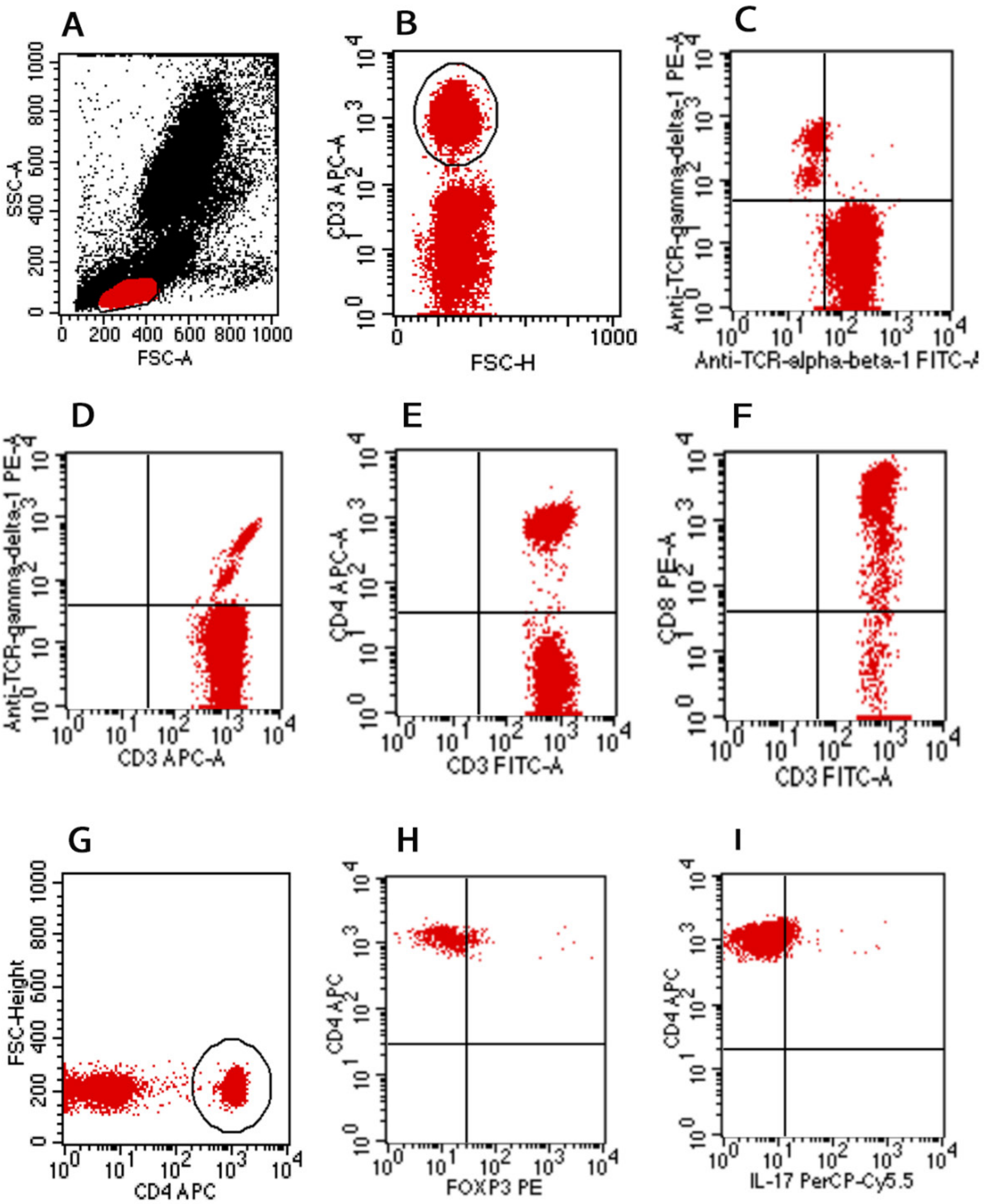

Figure S4. The data of the above pictures are from the COVID-19 recovered healthy people. **(A)** Lymphocytes were identified using forward and side scatter. **(B)** T lymphocytes were defined by the presence of CD3 and further divided into **(C-D)** αβ T cells, γδ T cells, **(E)** CD3+CD4+ helper/induced T cells, **(F)** CD3+CD8+ inhibited/cytotoxic T cells. **(G)**CD4+T lymphocytes were defined and further divided into **(H)** Tregs and **(I)** TH17 cells.

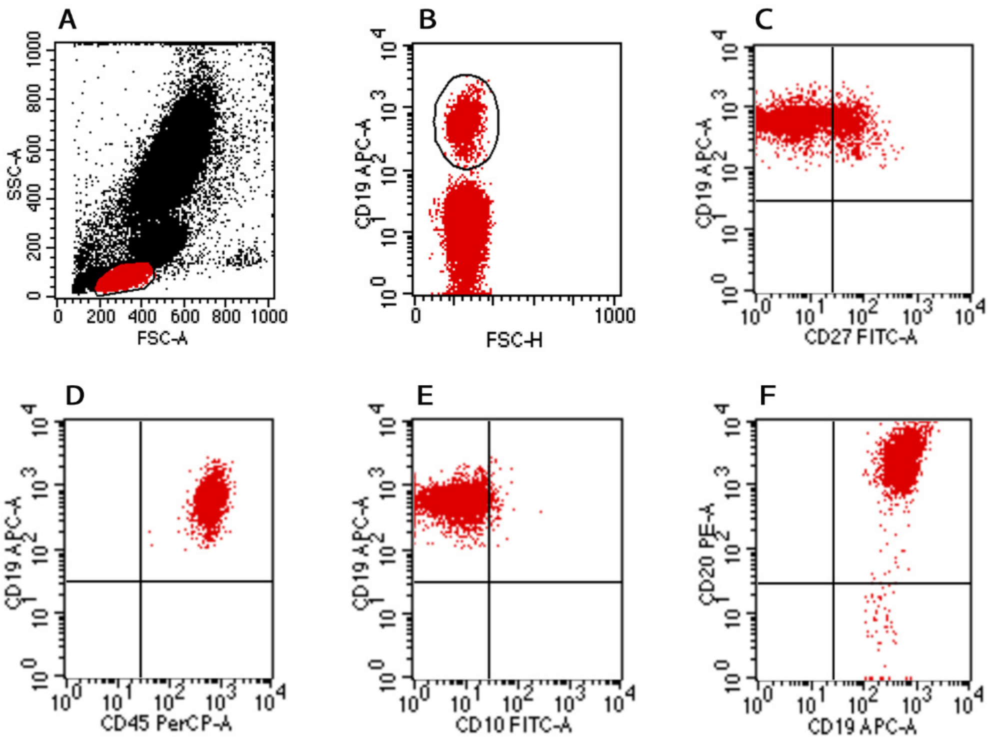

Figure S5. The data of the above pictures are from the normal uninfected healthy people. **(A)** Lymphocytes were identified using forward and side scatter. **(B)** B lymphocytes were defined by the presence of CD19, **(C-F)** and further divided into naïve B lymphocytes (CD10-CD19+ CD20+ CD27-CD38-/+ CD45+), memory B lymphocytes (CD10- CD19+ CD20+ CD27+ CD38-/+CD45+), transitional B lymphocytes (CD10dimCD19+CD20+CD27-CD38hiCD45+) subsets.

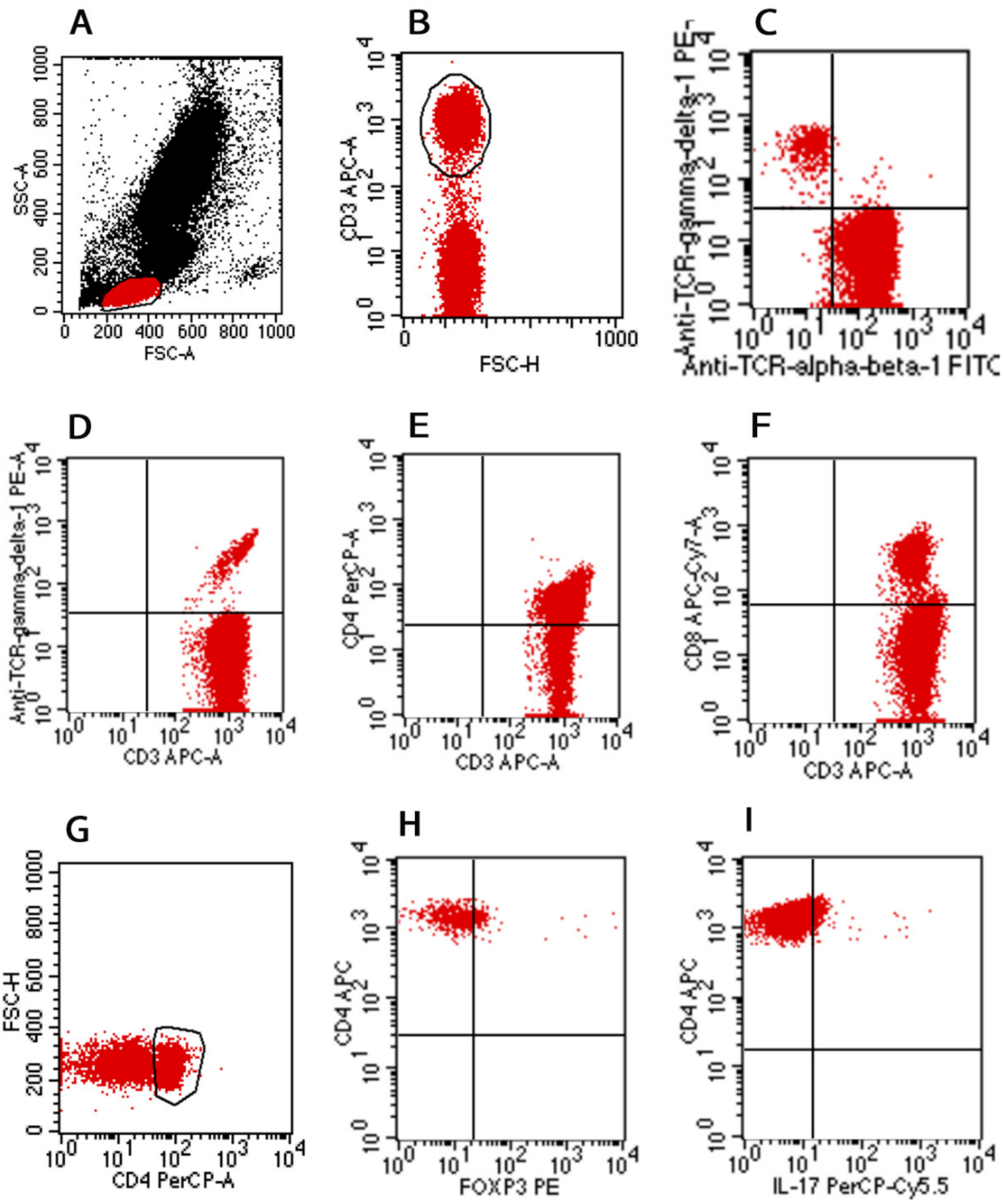

Figure S6. The data of the above pictures are from the normal uninfected healthy people. **(A)** Lymphocytes were identified using forward and side scatter. **(B)** T lymphocytes were defined by the presence of CD3 and further divided into **(C-D)** αβ T cells, γδ T cells, **(E)**CD3+CD4+ helper/induced T cells, **(F)** CD3+CD8+ inhibited/cytotoxic T cells. **(G)** CD4+T lymphocytes were defined and further divided into **(H)** Tregs and **(I)** TH17 cells.
